# Recent UK retirees’ views about the work-related factors which influenced their decision to retire: a qualitative study within the Health and Employment After Fifty (HEAF) cohort

**DOI:** 10.1186/s12889-022-12541-1

**Published:** 2022-01-17

**Authors:** Martin J. Stevens, Mary Barker, Elaine Dennison, E. Clare Harris, Cathy Linaker, Susie Weller, Karen Walker-Bone

**Affiliations:** 1 MRC Lifecourse Epidemiology Centre, Southampton General Hospital, University of Southampton, Tremona Road, Southampton, SO16 6YD UK; 2 MRC Versus Arthritis Centre for Musculoskeletal Health and Work, University of Southampton, Southampton, UK; 3 Clinical Ethics and Law at Southampton (CELS), Faculty of Medicine, University of Southampton, Southampton, UK

**Keywords:** Retirement, Work, Ageing

## Abstract

**Background:**

Lower birth rates and increasing longevity have resulted in ageing populations in European countries. These demographic changes place challenges on pension provision as numbers of those who are economically inactive and retired increase relative to those in paid work. Therefore, governments need workers to postpone retirement and work to older ages. Whilst health and wealth are important in retirement decision-making, considerably less is known about the effects of workplace factors. The aim of this study was to explore the views of recent UK retirees about the role that work-related factors played in their decision to retire.

**Methods:**

This qualitative study was nested within the Health and Employment After Fifty (HEAF) cohort. People who had retired 3-6 years previously (not for health reasons) were purposively sampled to obtain the views of men and women from a range of socio-economic backgrounds and jobs. Semi-structured interviews were carried out by telephone using a pre-defined topic guide. Interviews were audio-recorded, transcribed and analysed thematically.

**Results:**

Seventeen interviews were conducted. Thematic analysis showed that retirement decisions were complex and multi-factorial but that work-related factors contributed to decision-making in two main ways. First, some work factors pushed participants towards retirement. These were perceptions that: workplace change had affected the way they were valued or increased pressure on them; work demands, including commuting, had intruded excessively on personal time, effects that were exacerbated by modern technology; work was draining, isolating or under-appreciated; and /or that work was causing physical strain or discomfort relative to their perception of their capacity. In contrast, work factors could also cause participants to pull back towards work, particularly: autonomy; supportive work colleagues; a sense of being appreciated; and perceived job flexibility.

**Conclusions:**

Recent retirees explained that their decision to retire was multi-factorial but work-related factors contributed importantly. Potentially, employers could: review workers’ perceptions about their work; their capacity in relation to job demands; increase flexibility; and facilitate a supportive work community to encourage longer working lives.

## Background

The population of European countries has aged whilst birth rates have declined, re-shaping demographics. The Organisation for Economic Co-operation and Development (OECD) predicts that the old age to working age ratio (i.e. the ratio of people aged > 65 years /100 people aged 20-64 years), which was 13.9 people in 1950, will rise to 53.4 by 2050 [1]. Whilst life expectancy has increased, effective ages of retirement in OECD countries has decreased from the 1970s to early 2000s [2]. Although this trend has reversed somewhat since, the number of years that individuals spend in retirement has risen overall. This dual effect of increased longevity and earlier retirement has the potential to place strain on pension systems and the wider economy.

Consequently, most high-income countries have taken steps to encourage people to work to older ages [3] ‘a generalized shift from ‘pro-retirement’ to ‘pro-work” [4]. This includes a rise in the state pension age (SPA), which, in the UK, is in the process of increasing from 60 years (women) and 65 years (men) to 68 years for both sexes [5, 6]. This, along with the abolition of mandatory retirement in 2011 [7], formed part of a portfolio of changes aimed at encouraging working to older ages. It is important to acknowledge that retirement timing is not always at an individual’s discretion. Health is known to be a key factor [8–10], interestingly, the relationship between health and retirement is not straightforward, since people in good health, and particularly those in better socio-economic circumstances, may choose early retirement, perhaps because of a belief that health will inevitably decline and that they need to enjoy their health whilst they still have it [10–12]. Financial wellbeing is another important but complex factor since greater financial security may enable earlier retirement [4, 11] but, conversely, individuals with better incomes may also delay retirement [4, 11]. Even when people have planned their retirement, unanticipated events (such as disease or unemployment, partner ill-health, caring responsibilities) may impede them from acting according to plans [13]. Outside of unexpected events however, there is potential for increasing numbers of workers to be faced with more of a personal choice about when and how to exit paid employment.

Assuming good health and financial planning, individuals will consider a range of factors in planning the timing of their retirement, including work/life balance [14] and partner preferences. From the perspective of employers and policy-makers, however, work-related factors that influence retirement decisions could be a relatively straightforward target for interventions to encourage working to older ages. Therefore, as part of a mixed-methods retirement study, we conducted qualitative interviews with contemporary retirees in order to investigate what work-related factors had influenced their retirement decisions and what might have supported them to remain at work, whilst also permitting a wider exploration of factors influencing retirement that may not have been anticipated.

## Methods

### Sampling

This study was nested within the Health and Employment After Fifty (HEAF) study, a prospective cohort study incepted in England, UK, between 2013 and 2014 to investigate work and health amongst people aged 50-64 years (the detailed methodology is described elsewhere) [15]. Sampling was purposive, based upon socioeconomic status (defined by the National Statistics Socio-economic Classification three-class system [16]) and sex. Prior to 2018, state pension ages were different between men and women in the UK. Therefore our sampling decisions were taken to ensure that a wide variety of retirement experiences from across social strata were included in the study. For the current study, eligible HEAF participants comprised those who reported at baseline that they were ‘retired’, who had stopped working within the past 24 months and who had not left work for a health reason. Participants who were unemployed leading up to retirement were also excluded.

Eligible retirees were sent a participant information sheet with a consent form and pre-paid return envelope. Upon receipt of written consent, willing participants were contacted by telephone at a time convenient to them. Telephone interviews were chosen in order to keep the sample as inclusive as possible (participants live across England) and to make it as easy as possible for potential participants (telephone interviews have been shown to be less daunting and intrusive than in-person interviews [17]). There was no reward for, or inducement to, participate.

### Data collection

A topic guide was drawn up in advance, taking into account that the decision to retire is multifactorial [18] and that non-work factors are also relevant (Table 1). Initially, open questions were asked so as to allow participants to raise any issues pertinent to their personal retirement decision. The topic guide then focussed on the possible role of work factors in retirement, informed by findings from previous studies [9, 19, 20], and reflecting psychosocial work strain models [21–23]. Questioning covered: workload/effort; rewards, training and skills; control; environment; job satisfaction; and work community. The interviews continued until all topics had been addressed, however, questions were not fixed and topics could be addressed in any order depending on the course of the interview. The topic guide was tested with two practice telephone interviews with volunteers but no changes were required.

**Table 1:** HEAF FIRST topic guide

HEAF FIRST Topic guide	
Please note that the following questions were guides/prompts only. Fixed question were not asked. The topic guide was also considered and revised throughout the interview process.	
Topic	Example questions
**Retirement overview**	Would you describe yourself as retired? What does being retired mean to you? What age were you when you retired?
**Retirement decision**	What was the main reason for your retirement? What other reasons led to your retirement? What made the decision to retire more difficult?
**Workload/Effort**	How hard was your job physically? How hard was your job mentally? How important was your workload in your decision to retire?
**Control**	How much choice did you have in how you did your work? Prompt: could you decide when to take a break, could you decide what hours to keep, could you decide the best way in which to perform your role How much did you value that choice at work? How did the amount of control influence your decision to retire?
**Job Satisfaction**	How much did you enjoy your job? How important was job satisfaction in your decision to retire?
**Reward**	How well were you rewarded at your last job? What effect did the rewards have in your decision to retire?
**Work environment**	How much did your work change as you got nearer retirement? How much did work changes affect your decision to retire? How much say did you have in these changes?
**Training/skills**	How much training was available to you in your work? How much were your skills valued in your workplace?
**Community**	How was your relationship with your line manager? How important were your colleagues in dealing with work challenges? Did you retire earlier or later or at the same age as others at your workplace? If discrepancy – Was there a reason for the difference? How did your relationship with your colleagues affect your decision to retire?
**Employer interventions**	What could your organisation have done to encourage you to work for longer than you did?
**Wrap-up**	What else would you like to add about your retirement decision that we haven’t already covered?

Interviews were conducted by MJS, a 38-year-old man undertaking a PhD, trained in qualitative methods and interview skills, who had worked as research assistant on the HEAF study for 3 years. Consent was re-confirmed verbally before interviews commenced. MJS conducted the interviews in a private office and with participants’ consent, all were audio-recorded. Participants were usually at home and alone to the best of the researcher’s knowledge, although two interviews were interrupted and recommenced after a short break. MJS introduced himself and explained his role. Interviewees understood that MJS was not of retirement age, although this was never expressly stated. Field notes consisting of reflexive notes, details of interruptions and notes on the performance of the topic guide were recorded after each interview and if necessary were used to aid interpretation of the interview data.

### Data processing and analysis

Interviews were transcribed within 3 days by MJS. Transcripts were checked against recordings for accuracy but were not returned to the participants for comment. All identifying material was removed from the transcripts and pseudonyms used throughout. Data were analysed thematically [24] according to the methods of Braun and Clark. A critical realist epistemological stance was taken, as described by Maxwell [25]. Barbour [26] suggests that this is a *‘realist ontology (the belief that there is a real world that exists independently of our beliefs and constructions) with a constructivist epistemology (the belief that our knowledge of this world is inevitably our own construction created from a specific vantage point).’* Thematic analysis was conducted using Nvivo 11 [27] software.

Coding commenced alongside data collection, allowing monitoring of data saturation. Data from three of the interviews were independently and inductively coded by two authors (MJS and KWB) and results compared. A coding frame was developed which contained the code’s name, a description of the content and example quotes and was subsequently applied to all interviews. New codes were added to the coding frame as required and were described using illustrative quotations.

Candidate themes were derived from the data by grouping similar codes together. This process commenced after 13 interviews were fully coded to enable discussion with the wider research team. The candidate themes and updated coding frame were further tested by KWB and MJS through double-coding of three further interviews. Results were then compared, discrepancies discussed/resolved and candidate themes updated.

Interviews were conducted until saturation of themes relevant to the research question was attained. Saturation was defined as the point at which no new codes had been generated in three consecutive interviews for the range of possible work-related factors [28].

## Results

In total, 58 HEAF participants were contacted, of whom 18 (31%) agreed to participate. Only seventeen interviews were included in the analysis, however, since one participant reported unemployment prior to retirement. Participants retired between 2012 and 2014 at ages 55-67 years (therefore before, at, and after SPA) (Table 2). At the time of interview, in 2018, participants had been retired for between 3 and 6 years. The interviews lasted between 15 and 30 min, excluding introductions and post-interview conversations.

**Table 2:** Characteristics of participants in the HEAF FIRST qualitative study

	Men *N* = 8	Women *N* = 9	Total *N* = 17
Socio-economic status (SES)			
Higher and managerial	2	3	5
Intermediate	4	3	7
Routine and manual	2	3	5
Employment status prior to retirement			
Self-Employed (mean age at retirement 62.7 years)	2	4	6
Employed (mean age at retirement 60.9 years)	6	5	11
Retirement timing			
Before state pension age	5	2	7
At state pension age	2	3	5
Later than state pension age	1	4	5

**Fig. 1:**
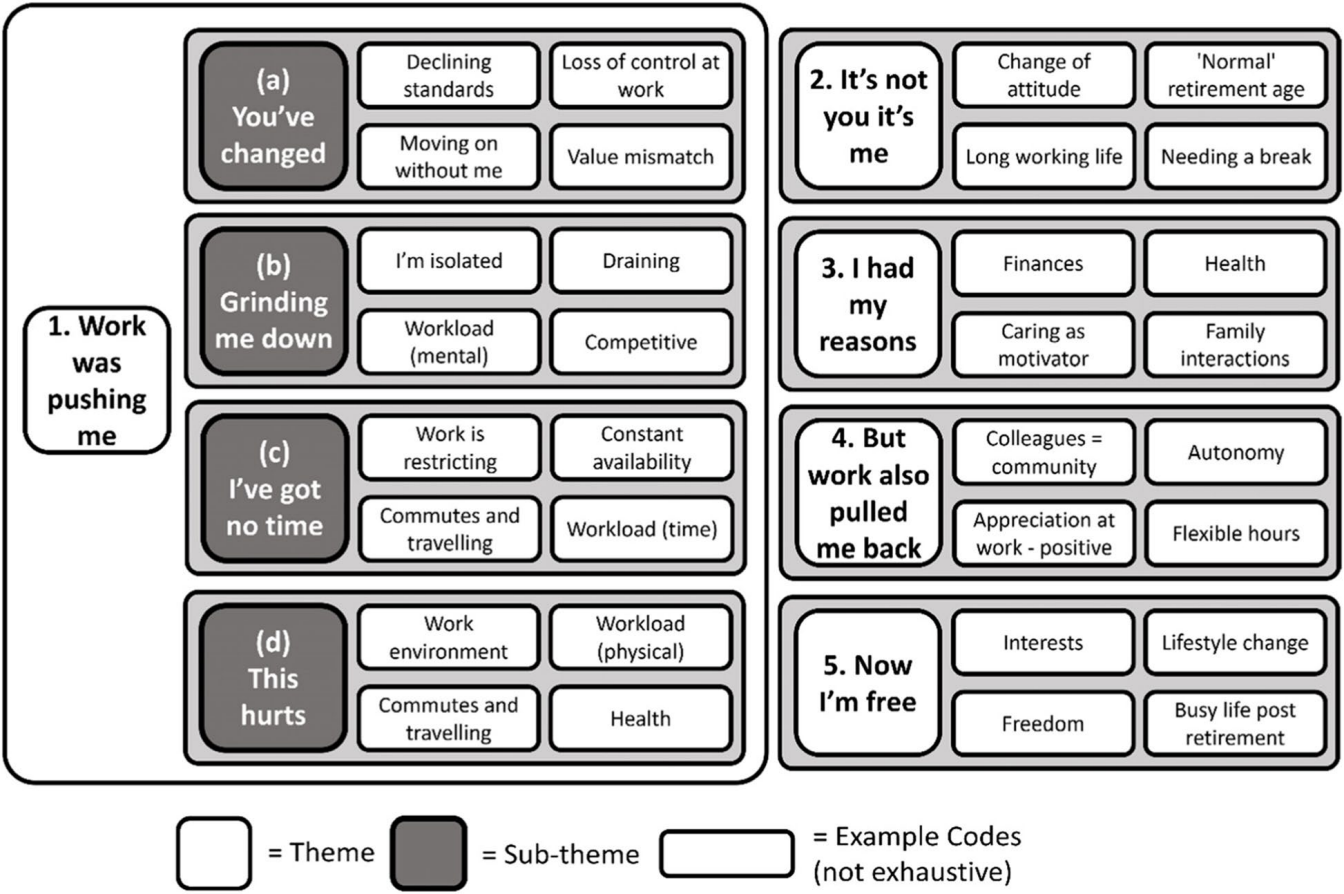
Summary of the codes and themes derived after thematic analysis of the 17 interviews

**Fig. 2:**
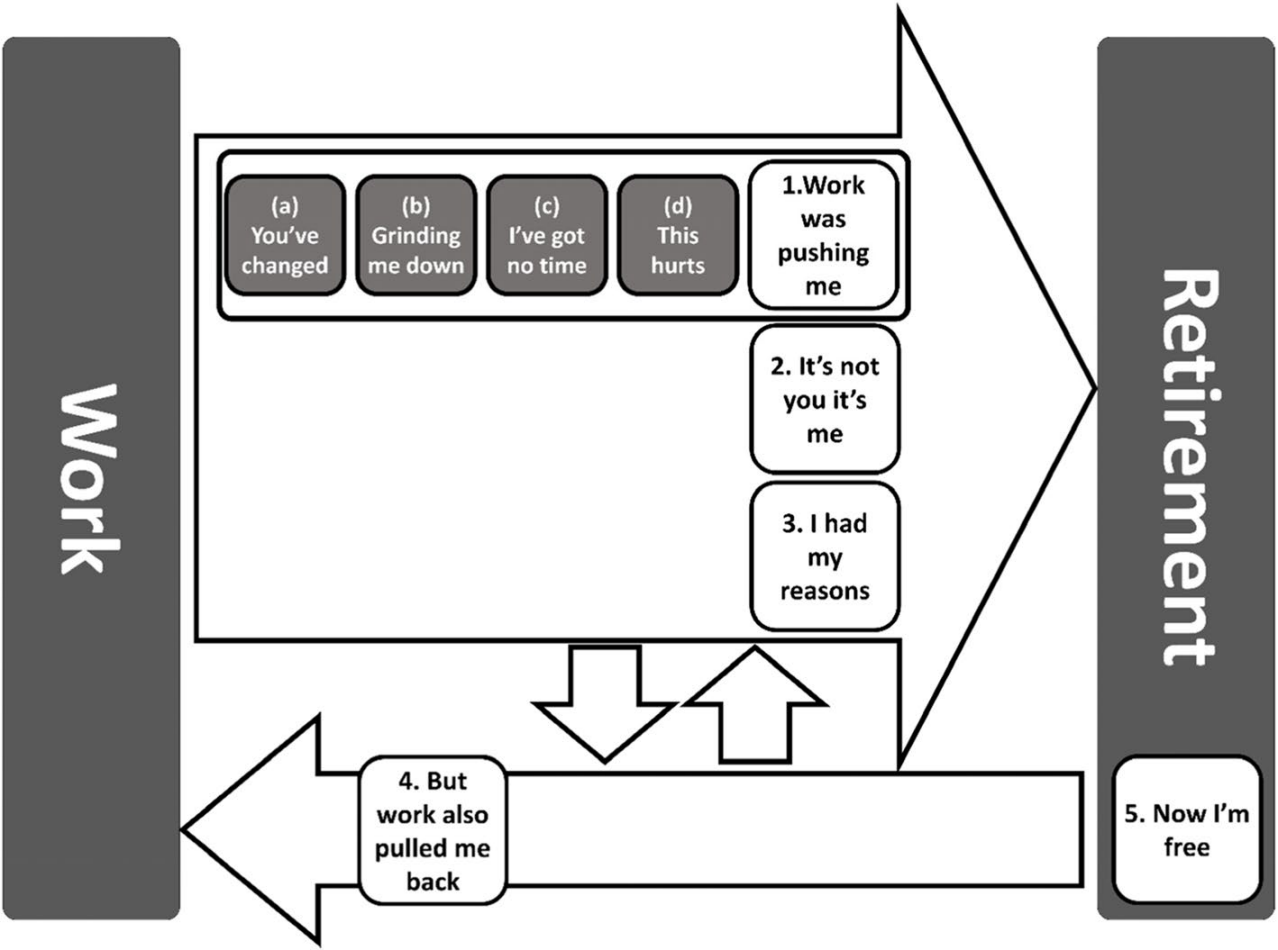
Thematic map of the HEAF FIRST qualitative study

Participants’ responses were organised into 44 codes and five themes (Fig. 1). Three of these themes, entitled 1:'Work was pushing me’ 2:'It’s not you it’s me’, and 3:'I had my reasons,’ described factors that pushed [29] the participant towards retirement (Fig. 2). 1:'Work was pushing me’ captured work-related factors that participants felt had pushed them to retire and was divided into a further four sub-themes. Theme 4: ‘But work also pulled me back’ included work factors that participants described as discouraging retirement. Theme 5: ‘Now I’m free’ covered perceptions of life in retirement.

Since our aim was to elucidate the work-related factors that affected retirement decision-making, the following section focuses on the themes 1:'Work was pushing me’ and 4:'But work also pulled me back’.

### Work was pushing me

Participants described a range of workplace factors that they viewed as having ‘pushed’ them towards retirement. These ‘push’ aspects were negative and/or unpleasant and participants described retirement as allowing them to remedy or escape these effects. This theme consisted of four sub-themes described below:

### 1(a) You’ve changed

Within this sub-theme, we grouped instances of where participants described workplace changes precipitating negative feelings. In some cases, workplace changes resulted in a new work environment, conditions or processes that retirees contrasted unfavourably with previous circumstances. Lisa said that she felt change had made her less valued. When asked what had changed, she replied:

*'I think the focus on the service changed. It was not anymore about giving a gold-standard service. In my opinion it was about delivering… volume'*

Like Lisa, some participants felt that workplace changes resulted in declining standards and/or increasing workloads, which conflicted with personal values or work-related pride, causing an imbalance for which the only solution was perceived to be retirement. Philip stated that upcoming changes to his workplace (a school) were his main reason for retirement. When asked to describe those changes, he said:

*'The sort of changes were cutback in financial support for the services we were providing and not being able to do the job that we… were employed for originally and it was being imposed upon us and it wasn't good for the children.'*

However, change was not always perceived negatively. When summarising his experiences near the end of an interview, Gareth said:

*'I love new things and challenges but you want to know that you have a secure position within that.'*

### 1(b) grinding me down

In this sub-theme, participants described aspects of their work that had become less bearable, (e.g. draining and competitive with heavy workloads) which had gradually pushed participants towards retirement. When asked if he enjoyed his job, Jamie replied:

*'Yes I would overall... until, as I say, the point it got a bit much… it was repetitive like that and the complaints got worse… over the years as well and the number of them, volume of them.'*

Within this sub-theme, participants perceived a lack of appreciation for their efforts. When asked whether her skills were valued in the workplace, Elena replied:

*'Well I think everyone sort of appreciated to a certain extent that actually you were doing a good job with the best you could, or the other staff did but, as much as appreciation from the hierarchy well… some of them stayed in their offices they didn't really get involved too much.'*

This sub-theme also included perceptions of being isolated at work. When Jamie was asked what he disliked about his job, he replied:

*'Well there was a certain amount of pressure. I was the only one in the office sometimes. You had to deal with everything that came up… and that led to the stress… There wasn't a lot of backup.'*

### 1(c) I’ve got no time

In this sub-theme, participants described work as a time-burden or restriction, which caused fatigue and prevented them from engaging in other more meaningful activities. Participants felt that excessive/increased hours infringed upon their personal time. When asked about her main reason for retirement, Betty said:

*'I was working a six-day week, not normal hours and it was usually a minimum of 60 hours… sometimes more… To be honest I don't want to work those sort of hours when I'm in my 60s.'*

Additionally, within this sub theme, commuting to and/ or from work was regarded as an increasing burden as the participant neared retirement. Gerard’s commute made him question his role:

*'There was a lot of driving time involved and as I say… the fact that I was spending what seemed to be an inordinate amount of time traveling, that was one of the reasons for saying, 'well why am I doing this?”*

Communication technology seemed, to some, to represent a growing burden, such that participants felt they were effectively constantly available for work, which further encroached upon personal time. Retirement seemed to offer alleviation from these restrictions. Julian described the moment he chose to retire:

*'I was at the hospital waiting to go into the operation and I was dealing with e-mails and phone calls and it suddenly struck me that this was not a way... that I wanted to live the rest of my life.'*

### 1(d) this hurts

Within this sub-theme, participants reflected on the relationship between physical comfort and work. Physical workloads that were previously normal became more unpleasant and unduly burdensome, as the participant neared retirement, sometimes even if the work was otherwise enjoyable. When asked to summarise her retirement decision, Louisa responded:

*'I think it's got to be summed up in one word: pain. I was fed up with the pain… although I had so much good things, there were so many good things about it, the pain overrode everything.'*

In general, the mismatch between physical work demands and declining physical abilities was described as a personal shortcoming by participants, rather than an imbalance that could be potentially rectified through work modification. In some cases, the physical work environment (unhealthy, unpleasant) pushed individuals towards retirement. Leo was asked whether physical workload influenced his decision to retire, he responded:

*'I always felt that obviously doing a lot of heavy lifting and pulling and pushing, sledge-hammering was partly good because it kept me fit but at the same time…, as I got older things were making me a bit out of breath and I could see it was making me, bit unhealthy at the same time. So I decided to knock it on the head.'*

### But work also pulled me back

In contrast, we identified a theme in which work-related factors appeared to weigh against the decision to retire. These factors acted as counters to the more negative ‘push’ aspects of work described above. Participants described work as providing many positives, including a sense of pride and status. Some reported perceiving work as a break from the rigours of outside life. Participants reported that having supportive colleagues formed a community both in and outside work. Loyalty to this community and unease at the prospect of leaving it seemed to make retiring more difficult. When discussing colleagues’ influence on her retirement decision, Alice said:

*'I didn't feel I could just walk away. That wouldn't be fair to somebody you worked with for 25 years.'*

This theme also captured opportunities for the negative ‘push’ aspects of work to be mitigated e.g. by having autonomy and/or being appreciated. Greg, a factory worker, described his workplace as increasingly complex. When asked whether this had influenced his retirement decision, he replied:

*'No, because basically the involvement I had from, well day one, meant I was part of the process.'*

Some participants described how flexibility in working hours acted as a pull towards work. Patsy described a situation where her partner had become seriously ill:

*'I had time off and if I wanted to I could've worked from home and just when he came out of hospital. Things like that, they were… very good. But I managed to go back after about two or three weeks on a part-time basis and then went back full-time later on.'*

### Other themes

Two other themes included factors that pushed participants towards retirement but were notionally unrelated to the workplace. In theme 2:'It’s not you it’s me’, the push towards retirement came from personal values or feelings, rather than being generated by external circumstances. For example, participants suggested that there was a ‘normal’ age to retire or a point at which retirement was almost inevitable. In theme 3:'I had my reasons’, the main factors pushing towards retirement seemed to be external to the participant or were outside of their control. These factors acted upon the participant and changed their plans. In particular, this theme captured the role of factors such as: health; finances; caring responsibilities; and family interactions in the timing of retirement and, for these individuals, work factors took a lesser role. The fifth theme, ‘Now I’m free’, encompassed descriptions and perceptions of life in retirement. Retirement was often described in terms of freedom, which was a counterpoint to the restrictions of their job, providing an escape from the negative aspects of work or other push factors.

## Discussion

Using qualitative methods, we researched the views of men and women, employed in a range of jobs (including for example manufacturing, health and social care, retail and civil service employees) about their decision to retire 3-6 years earlier (excluding those who retired for health reasons). We invited participants to tell their own stories and contextualise their retirement decisions by describing every aspect. This was important because for the most part, the decision to retire was multi-factorial, suggesting an interaction of, for example, perceived health and workplace changes. Consequently, as expected, some of the reasons participants gave for retirement were not work-related. We found considerable evidence, however, that work-related factors played a role in participants’ decision-making and that these influences acted to both ‘push’ them away from work or ‘pull’ them back towards it.

Participants gave examples of how potentially negative aspects of their work could be effectively countered by positive features, even though participants eventually all decided to retire. For example, workplace change appeared to have contributed importantly to several retirees’ decisions, particularly when changes were perceived as leading to declining standards or higher work demands. Equally, however, a participant who felt fully involved in workplace change explained that the change had not pushed him towards retirement because his employers had engaged him in the change process. In some cases, participants seemed to become less able to tolerate aspects of their job (e.g. working in isolation) so that they felt it ‘grind them down’ and push them away. It seemed the retirees became less inclined to accept work-related time-burdens as they neared retirement, so that excessive hours, long commutes or being available out of hours became more of a ‘push’ to retire. It is possible that reporting of some of these factors in this way is part of the so-called decline narrative or ‘health pessimism’ [10], reflecting a societal belief that ageing is associated with declining health and that retirees need to avoid running out of time to make maximal use of their best health by retiring promptly. Researchers have recently pointed to the importance of the role of such internalized age stereotypes and norms in inhibiting older workers [30]. Whilst this was not our specific focus, it is important to bear in mind that some participants’ perceptions of the role of work factors in their retirement might derive from age perceptions that have been normalised within the organisation in which they were working. Certainly this appeared to be the case with the physical demands of the work, which, when coupled with a corresponding self-perceived decline in their resilience or capability, pushed individuals towards retirement. Interestingly, participants tended to speak about this as if they were personally to blame and therefore suggests that they were to some extent influenced by the age stereotypes, expecting their older age to be accompanied by poorer functional capability. In mitigation, however, retirees described several factors that pulled them to continue working, such as supportive relationships with colleagues, a feeling of being appreciated, and flexible hours.

Our finding that physically-demanding work was more difficult to sustain at older ages was previously reported in another qualitative study undertaken in the Netherlands [20]. Interestingly, however, a number of quantitative studies exploring the same question have been inconclusive [18, 31–33]. Our data do however highlight that the physical nature of an individual’s work only became important to retirement decisions when accompanied by a corresponding perceived decline in physical capabilities. Numerous retirement studies ascertain the frequency, intensity and duration of physical work demands, but it may be more relevant to understand how older workers perceive they are coping with the physical demands of their work and thus explore any change in an individual’s capacity or resilience to meet these work demands. This also has implications for longitudinal studies that measure self-reported workload at baseline as a risk factor for retirement several years subsequently. If, as this study suggests, it is not the demands of work itself but the change in capacity or resilience to meet the demands that matters, then the point may be being missed. If this hypothesis stands up, employers seeking to retain older workers may be advised to introduce regular performance or career reviews with older workers to take into account their perceptions about the match between their changing physical capacity and the work demands. Muscle mass and strength are known to peak at around 30-40 years of age [34] after which there is a general decline. Whilst experience can mitigate these effects, it is unrealistic to expect older workers to retain exactly the same capacity throughout their working life. Employers and policy-makers need to consider options for redeployment or re-training or be prepared to accommodate the changes in physical function and resilience of older workers.

That older workers begin to resent the incursion of their work on their personal time more as they near retirement is perhaps not unexpected but is important. Sewdas [35] previously identified flexible working practices as a pre-condition for maintaining longer working lives and flexibility is probably the solution which best enables the individual to balance changing priorities. Our study highlights commuting times, long working hours, working in isolation and an expectation that workers should be available out of hours as some key issues that employers could address using innovative solutions to reduce the perceived incursion on personal time, thereby enabling prolonged working.

Our data demonstrate that retirement decisions are multifactorial in line with prior studies [20]. Financial considerations and health were reported by participants as relevant to their retirement decisions. Our focus on work factors was chosen to better understand what, in the context of people’s health and financial circumstances, might be modified to encourage working to older ages. Our findings of the importance of factors such as: having supportive working colleagues; autonomy in the workplace; flexible hours; and appreciation at work highlights areas in which employers could focus attention in order to retain older workers. Importantly however, these factors are known to be important amongst workers of all ages and have been captured by a variety of different models of work stress [21, 22]. Work stress is associated with impaired mental and physical health cross-sectionally and longitudinally [36–38] and measures to reduce these impacts should be encouraged for workers of all ages, rather than singling out older workers alone for such initiatives, which may encourage age discrimination and may be perceived by the workers themselves as devaluing them as a group, as suggested in prior research [39].

Our findings need to be considered alongside some limitations. The participants had retired some 3-6 years ago. It is feasible that recollections of retirement decisions may have changed over time and that they may have reported different determinants had we been able to interview them contemporaneously. It is not clear whether the passing of time would make perceptions of former workplaces more, or less, favourable. Although participants generally reported enjoying their retirement, they still had fond recollections of work, despite frustrations which had contributed to their retirement. Importantly, as they had all chosen to retire, the factors reported as pulling participants back towards work had not persuaded them to continue working. It may not necessarily be possible to extrapolate our findings to adults working beyond SPA. However, 5 of the 17 participants in the current study had worked beyond their SPA, so the factors reported here were at least of some relevance to longer working lives. Member-checking of transcripts for trustworthiness was not feasible within the time-frame of this project because the findings were needed to inform the design and content of a questionnaire for a subsequent case-control study. It is impossible to know if our results would have differed substantially if this step had been feasible. We chose a priori to sample from amongst people who reported that their retirement was not for health reasons. This was to enable us to better understand the role of work factors amongst people whose health was good and therefore might be expected to have some choice about timing of retirement rather than those who may have been forced to retire for a health reason. However, the effect of this may have been to exclude those people who were most struggling with the physical demands of their role.

The invitation explained that the interview would explore retirement decisions and work conditions prior to retirement and it is possible that those who had retired for work-related reasons may have been more likely to respond. However, the overall sampling frame of the HEAF study was from general practice registers, recognised to be almost exactly representative of the general population. Furthermore, retirement was not emphasised in the original HEAF study aims.

Although interviews were continued until all topics had been addressed, it is possible that the duration may not have been long enough for in-depth reasons for retirement to be revealed. However, it may also be the case that for some participants, reasons for retirement were relatively simple and could be expressed succinctly.

Further, although we are confident that data saturation was reached in the terms adopted by our study, it is possible that further interviews may have revealed more, less commonplace reasons for retirement. Therefore our study cannot be considered an exhaustive list of reasons for retirement.

Our study has particular strengths. Our participants are among the first individuals in the UK who retired after the implementation of government measures, including increase in the SPA and abolition of mandatory retirement, intended to encourage working to older ages. Women participating in this study were also subject to incremental changes in the SPA [5] which were rolled-out throughout the period during which they made their retirement decisions. Therefore, our study explored a wide range of work and retirement factors that individuals viewed as important to them when making voluntary retirement decisions in a contemporary context. Furthermore, the HEAF study includes inhabitants from all over England and from all 10 deciles of deprivation and we chose to purposively sample women and men from across the socio-economic spectrum, and additionally included individuals who retired before, at, and, (nearly 30% of the sample) after SPA.

## Conclusions

Amongst recently-retired English workers, the interaction of work and personal factors along with the individual’s own perceptions were decisive in the balance of pushes and pulls that tipped the participants towards their retirement decision. The work factors identified could be addressed by practical interventions to support people who wish to continue working to do so in comfort and good health, potentially lengthening working lives. This qualitative work has also highlighted new areas of questioning that could be explored in qualitative or quantitative retirement studies. We will investigate these findings further in a subsequent case-control study on retirement decisions which will also be nested within the HEAF cohort.

## Data Availability

The dataset generated and analysed during the current study are available from MJS but restrictions apply to the availability of these data, which were used under license for the current study, and so are not publicly available. Data are however available from the authors upon reasonable request and with permission of KWB.

## Abbreviations

HEAF: The Health and employment after 50 study
SPA: State pension age
